# Quantum Nanomedicine and Quantum Biomaterials

**DOI:** 10.1002/advs.75557

**Published:** 2026-05-06

**Authors:** Xinyue Dai, Liang Chen, Wei Feng, Yu Chen

**Affiliations:** ^1^ Materdicine Lab School of Life Sciences Shanghai University Shanghai China; ^2^ Shanghai Institute of Medicine Shanghai China

**Keywords:** quantum biology, quantum biomaterials, quantum medicine, quantum nanomedicine

## Abstract

Quantum nanomedicine and quantum biomaterials, as an interdisciplinary field, deeply integrate quantum science, material science, nanotechnology, biology, and medicine. Here, we define quantum nanomedicine and quantum biomaterials as a paradigm that harnesses quantum effects in nanomedicine and biomaterials, including quantum superposition, quantum coherence, quantum tunneling, topological quantum effects, and spin polarization, to achieve either spatiotemporally precise modulation of physiological activity and therapeutic intervention or the enhancement of intrinsic physiochemical properties for amplified therapeutic outcomes. This article systematically elucidates how quantum effects govern fundamental life processes and prospectively explores their potential in enabling innovative therapeutic strategies. The typical mechanisms of quantum biological effects involve quantum coherence in photosynthetic energy transfer, spin polarization in modulating reactive oxygen species generation, and quantum biological electron tunneling as verified in cytochrome c (Cyt c). These principles provide a theoretical foundation for the rational design of quantum nanomedicine and biomaterials. By controlling quantum coherence, quantum tunneling, and spin properties, the precise spatiotemporal regulation of biomolecular interactions and cellular signaling pathways can be achieved. The herein proposed quantum nanomedicine and quantum biomaterials establish a new paradigm for intervening in life processes at electronic and informational levels, thereby laying a scientific foundation for developing next‑generation diagnostic and therapeutic platforms.

## Graphical abstract

In this perspective, we define quantum nanomedicine and quantum biomaterials as a paradigm that harnesses quantum effects in nanomedicine and biomaterials, including quantum superposition, quantum coherence, quantum tunneling, topological quantum effects, and spin polarization, to achieve either spatiotemporally precise modulation of physiological activity and therapeutic intervention or the enhancement of intrinsic physiochemical properties for amplified therapeutic outcomes.

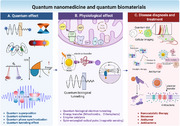

Quantum mechanics, a foundational framework for describing microscopic phenomena, has not only redefined our conception of matter but has also provided transformative conceptual and methodological tools that propel ongoing advances across chemistry, biology, and information science [1]. A growing realization is that quantum effects are deeply embedded in the fundamental mechanisms that drive biological processes and sustain life [2, 3, 4]. This is exemplified by quantum‐assisted electron transfer, a process facilitated by proteins either enzymatically or via superexchange tunneling pathways. By enabling efficient catalysis via low‐energy transition states, it underpins vital biological processes including photosynthesis, respiration, and DNA repair. Particularly, photosynthesis, as a highly optimized energy conversion system in nature, achieves energy transfer efficiency approaching the quantum theoretical limit, serving as a key paradigm for investigating quantum effects in biological systems [4]. In the phycobilisome complexes of red algae and cyanobacteria, allophycocyanin serves as the core light‐harvesting antenna, responsible for receiving light energy from the phycobilisome rods and directing it to the reaction centers of photosystems with an overall quantum efficiency exceeding 90%. The ability of migratory animals, such as birds and fish, to navigate by sensing the Earth's magnetic field stands as another classic example of quantum coherence in biology. The mechanism involves light‐induced generation of a pair of radicals with entangled spins, whose chemical reaction rates are influenced by weak magnetic fields, thereby providing the organism with a magnetosensory signal. In enzymatic catalysis, electrons are rapidly transferred between active sites via tunneling through the protein medium, a process that operates with significantly higher efficiency than classical diffusion mechanisms [2].

This inquiry raises essential questions concerning the operational mechanisms of biological systems, specifically whether natural energy transfer processes genuinely utilize quantum coherence to achieve directional regulation. If confirmed, a further question arises as to how such subtle quantum phenomena can be effectively sustained and integrated within the highly dynamic microenvironments of living organisms. Additionally, it remains to be examined how quantum chemical principles, such as electron tunneling, coherent superposition, and quantum entanglement, actively participate in regulating life processes at molecular and cellular scales. Investigating these questions not only deepens our understanding of bioenergetic mechanisms but also promotes interdisciplinary convergence among quantum physics, chemistry, and biology. The nanoscale (1–100 nm) serves as a transitional regime between classical and quantum physics. When material dimensions are reduced to the nanometer range, the confinement of electron wavefunctions by boundaries causes the continuous band structure to break into discrete energy levels, giving rise to the quantum confinement effect. The strong quantum confinement in quantum dots enables precise control over their photoluminescence maxima through size variation, a property that has been effectively harnessed in diverse bioimaging modalities.

Building on the arguments presented, we herein for the first time define quantum nanomedicine and quantum biomaterials as a paradigm that harnesses quantum effects in nanomedicine and biomaterials, including quantum superposition, quantum coherence, quantum tunneling, topological quantum effects, and spin polarization, to achieve either spatiotemporally precise modulation of physiological activity and therapeutic intervention, or the enhancement of intrinsic physiochemical properties for amplified therapeutic outcomes. To further differentiate the field, we propose a conceptual hierarchy that separates fundamental quantum chemical principles from engineerable emerging quantum effects. Fundamental principles, such as superposition in molecular orbitals and quantum phase synchronization, are common to all molecular systems and thus cannot uniquely define quantum nanomedicine. In contrast, this field centers on the deliberate engineering of emerging phenomena, for example, quantum coherence, spin‐related effects (spin polarization and the spin Seebeck effect) [5], and topological states (Dirac or Weyl semimetals). Quantum confinement acts as a bridging mechanism that actively harnesses size‐tunable properties for biomedical intervention. Quantum nanomedicine is defined not by the mere presence of quantum mechanics, but by the strategic manipulation of non‐trivial quantum effects to achieve targeted therapeutic gain (Figure 1). It should be noted that quantum biology focuses on naturally occurring quantum effects in biological systems (e.g., avian magnetoreception, photosynthesis) without external engineering. Conventional nanomedicine relies on size‐dependent classical properties (e.g., enhanced permeability and retention (EPR) effect, high surface area) to improve drug delivery and diagnosis. In contrast, quantum nanomedicine is defined by the intentional engineering of quantum‐confined states, such as tunable electronic structures, spin polarization, and quantum coherence, to actively trigger biological responses or enhance therapy. Thus, quantum nanomedicine is not an extension of classical nanomedicine nor a passive observation of quantum biology, but an intervention‐oriented discipline that harnesses non‐trivial quantum effects for targeted therapeutic gain.

**FIGURE 1:**
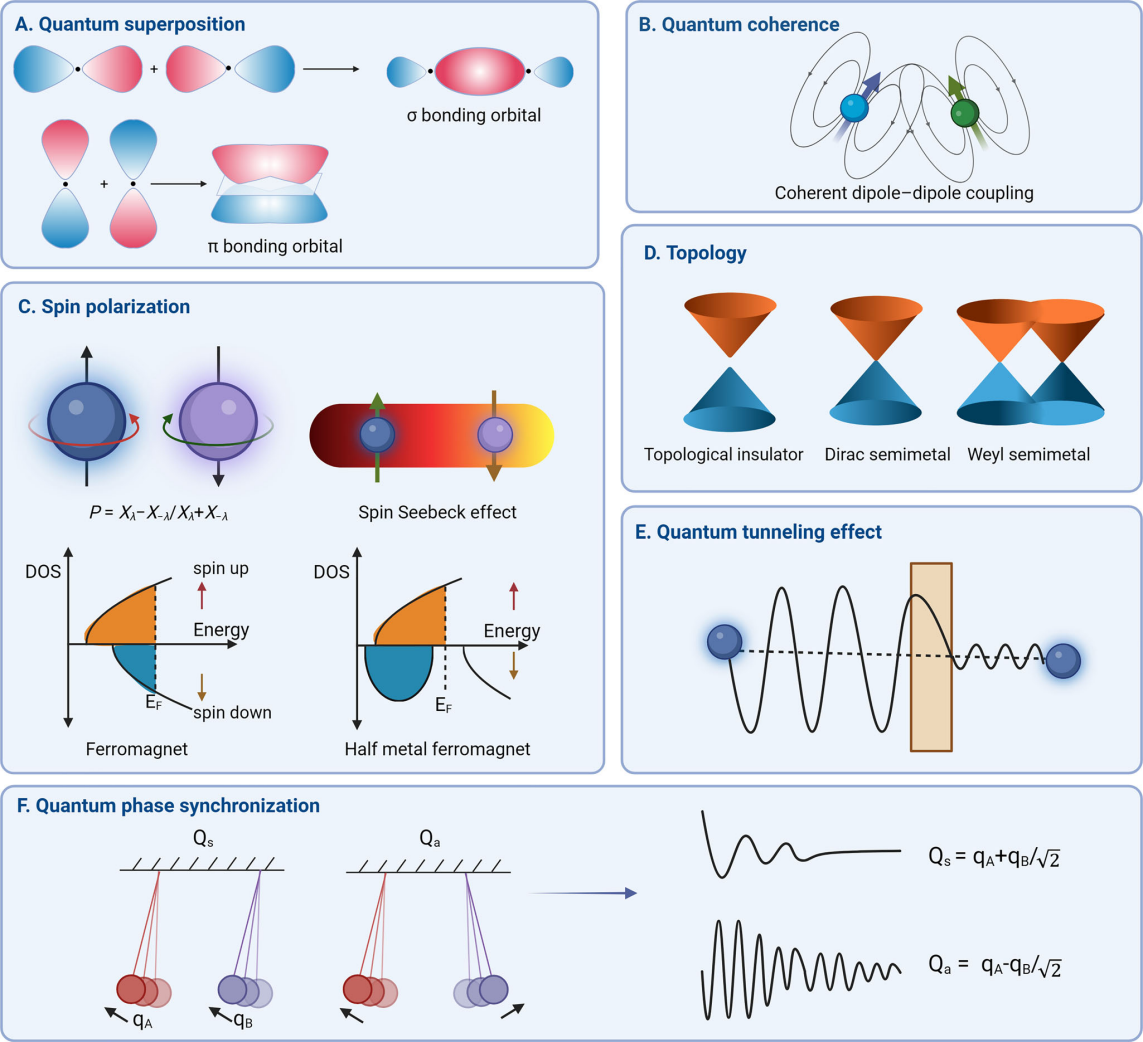
Schematic illustration depicting quantum mechanics‐related concepts. (A) Superposition of quantum states and its manifestation in σ‐bonding and π‐bonding configurations. (B) Quantum coherence phenomena, exemplified by coherent dipole‐dipole coupling in interacting quantum systems. (C) Spin‐related quantum effects in spintronic systems encompass a range of phenomena, including spin polarization, the spin Seebeck effect, as well as distinctive material classes such as half‐metallic ferromagnets that exhibit complete spin polarization at the Fermi level. (D) Topological insulators featuring protected surface states, along with relativistic Dirac and Weyl semimetals. (E) Quantum tunneling effect. (F) Quantum phase synchronization. Created in https://BioRender.com.

Each quantum phenomenon uniquely controls specific physiological processes, forming the basis for biomedical translation. Quantum superposition determines the electronic architecture and catalytic reactivity of nanomaterials, offering a direct design basis for pancatalytic medicine. Quantum coherence enables directional energy transport via coherent dipole‐dipole coupling, which can enhance photodynamic or photothermal energy transfer. Spin‐correlated effects provide distinct mechanisms for regulating radical generation and thermoelectric catalysis, respectively. Based on this framework, these fundamental quantum‐mechanical concepts drive a cascade of physiological processes and underpin innovative strategies for disease diagnosis and treatment (Figure 2). At the molecular level, the superposition of quantum states manifests in the formation of σ‐ and π‐bonds through the linear combination of atomic orbitals, defining chemical bonding and reactivity. These phenomena directly underpin the emerging discipline of nanocatalytic medicine, as nanocatalytic medicine precisely involves designing and modulating the electronic structures, surface states, and band characteristics of nanomaterials (such as quantum dots and metal nanoclusters) to activate or enhance their catalytic reactions within biological environments [6]. In energy transfer processes, quantum coherence, which is exemplified by coherent dipole–dipole coupling, enables highly efficient energy transport in systems such as photosynthetic complexes [7, 8]. The spin degree of freedom gives rise to phenomena including spin polarization, spin Seebeck effect, and fully spin‐polarized half‐metallic ferromagnets. Spin polarization can modulate the efficiency and selectivity of reactive oxygen species (ROS) generation [9, 10], thereby optimizing the therapeutic outcomes of nanocatalytic therapy. For example, enhancing sonocatalytic therapy via electronic spin states has been demonstrated [10]. Spin polarization reconfigures the electronic structure and boosts the density of spin‐polarized electronic states. As a result, reactant adsorption energy is lowered, charge transfer efficiency is increased, and both intrinsic catalytic activity and ROS production are enhanced for effective tumor treatment. In thermoelectric catalytic therapy, the spin Seebeck effect enhances the local electric field to improve energy conversion efficiency and promote more effective catalytic reactions [11, 12]. Furthermore, advanced materials such as fully spin‐polarized half‐metallic ferromagnets can be utilized to construct intelligent catalytic systems responsive to magnetic fields or temperature changes, demonstrating significant potential in smart medical devices and precision therapy.

**FIGURE 2:**
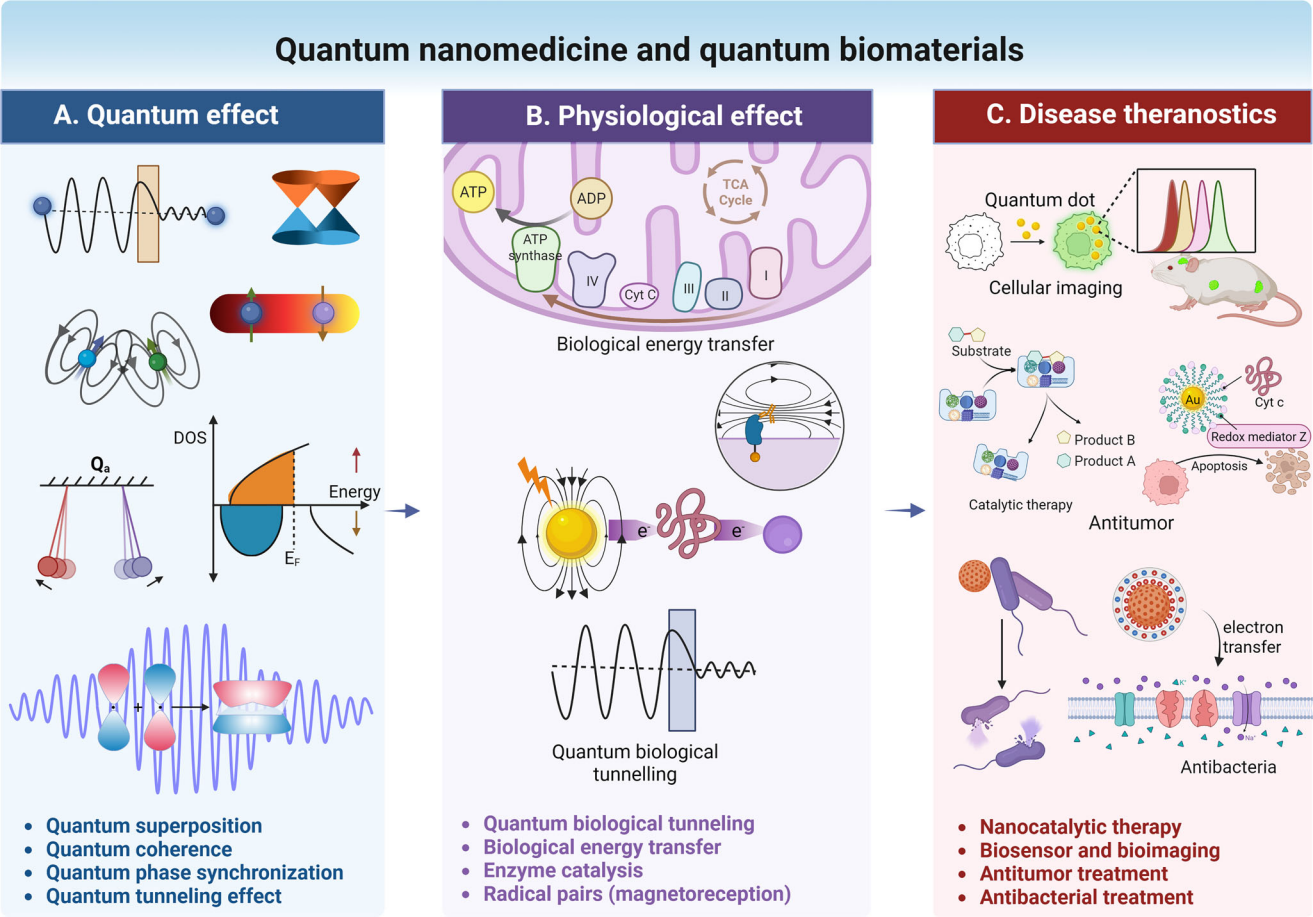
Overview of quantum nanomedicine and quantum biomaterials. (A) Engineered quantum effects in nanomaterials, including quantum coherence, tunneling, and spin polarization. (B) Quantum phenomena in physiological processes are governed by these quantum effects. (C) Corresponding applications in disease diagnosis and therapy, leveraging engineered quantum effects for precise and amplified medical interventions. Created in https://BioRender.com.

Topological quantum states, such as those in topological insulators and Dirac/Weyl semimetals, exhibit protected surface states and relativistic electronic dispersions governed by band topology. The impact of this phenomenon on catalysis has been extensively reported [13, 14, 15]. Topological quantum materials, represented by topological insulators, possess topologically protected surface states that offer high‑density active sites, excellent electron‑transport capability, and robust environmental stability, thereby significantly enhancing catalytic efficiency and spatiotemporal controllability. These properties endow them with considerable potential in nanocatalytic medicine, providing a theoretical foundation for developing novel intelligent catalytic therapy platforms. Further fundamental quantum effects include quantum tunneling, which facilitates particle transmission through classically forbidden barriers, and quantum phase synchronization, which enables coherent dynamics in coupled quantum systems. The observation of quantum biological electron tunneling in living Cyt c further verifies the involvement of quantum coherent mechanisms in intracellular electron transfer processes [2]. The mechanism by which bio‑nanoantennas constructed from covalent conjugation of gold nanoparticles with Cyt c induce cancer cell apoptosis through quantum signal transduction has been demonstrated [16]. These fundamental quantum‐mechanical concepts drive a cascade of physiological processes and underpin innovative strategies for disease diagnosis and treatment. The aforementioned research indicates that designing nanostructures and biomaterials with tailored quantum properties to precisely regulate biomolecular interactions, cellular signaling, and neural functions represents a strategic direction for next‐generation nanomedicine and biomaterials.

In the field of quantum biomaterials, quantum dots (QDs) stand as a quintessential manifestation of engineered quantum phenomena. Their distinct electronic and optical characteristics arise directly from quantum confinement effects, which allow for meticulous tuning of optoelectronic behavior by simply modulating particle size and composition. This capacity for precision control has propelled significant advances across a spectrum of disciplines, most notably in nanomedicine. For therapeutic applications, QDs function through mechanisms such as controlled photodynamic therapy (PDT) [17]. Under targeted light irradiation, QDs act as potent photosensitizers, simultaneously initiating two primary cytotoxic pathways: the generation of ROS (via type I/II PDT mechanisms) and the production of localized hyperthermia (through photothermal effects). Moreover, the inherent brightness, photostability, and multiplexing potential of QDs render them invaluable as high‐fidelity imaging probes and sensors, thereby bridging diagnostic and therapeutic modalities in emerging theranostic platforms. Beyond quantum dots, recent reports have documented the successful use of engineered quantum biomaterials for precise quantum sensing and remote modulation within living systems. These systems employ genetically encoded proteins (e.g., magneto‐sensitive fluorescent proteins MagLOV [18]) as structural scaffolds. Using genetically encoded scaffolds like MagLOV, these systems rely on endogenous flavin cofactors interacting with the protein backbone to form spin‐correlated radical pairs (SCRPs). As quantum active centers, the SCRPs exploit quantum spin dynamics to boost fluorescence signals, leading to improved multimodal sensing [19] or biological imaging [18].

The advancement of quantum nanomedicine and quantum biomaterials requires a convergent integration of quantum science, nanotechnology, and systems biology, moving beyond the observation of quantum biological phenomena toward their active manipulation. Leveraging nanobiomaterials to induce quantum effects and achieve quantum‐precision intervention in physiological and pathological signaling for disease diagnosis and treatment represents a highly promising frontier in modern medicine. This paradigm is grounded in nanoscale physical phenomena such as quantum coherence, quantum tunneling, and spin polarization, enabling precise intervention in biological processes at the energetic, electronic, and informational levels, with a degree of specificity and spatiotemporal resolution unattainable by conventional means. Notably, biological quantum tunneling effects inherently exist in fundamental life processes such as photosynthesis, respiration, DNA repair, and intercellular communication.

Experimentally validated quantum biological effects include quantum‐assisted electron tunneling in Cyt c, which facilitates intracellular electron transfer and has been harnessed via gold nanoparticle‐based nanoantennas to induce apoptosis. In parallel, the quantum confinement effect endows quantum dots with tunable photodynamic activity, and spin polarization has been demonstrated to regulate reactive oxygen species generation in nanocatalytic platforms. Building upon these mechanisms, quantum biomaterials are expected to enable increasingly precise subcellular interventions. Nevertheless, the application of externally coupled quantum materials for real‐time, closed‐loop modulation of organellar functions (such as mitochondrial ATP synthesis) remains a conceptual frontier. Achieving this capability will require overcoming substantial challenges, including the preservation of quantum states in vivo, organelle‐specific targeting, and the integration of real‐time quantum sensing with feedback control. From the perspective of nanomedicine, this strategy has already demonstrated potential in several key areas. For instance, the use of quantum dots or plasmonic excitation materials to enhance photodynamic efficacy and induce cancer cell apoptosis has been extensively investigated.

Nevertheless, the advancement of quantum nanomedicine and quantum biomaterials toward widespread research and practical implementation is constrained by several critical challenges:

1. **Maintaining the stability of quantum states in complex biological environments**. The fundamental prerequisite for achieving quantum biological regulation lies in preserving the coherence and stability of functional quantum states within living systems. Biological environments are intrinsically complex, where inherent thermal fluctuations, ionic interference, molecular collisions, and complex electromagnetic backgrounds can induce quantum decoherence, rapidly extinguishing delicate quantum effects. Therefore, it is imperative to develop multi‐pronged synergistic strategies to protect quantum information. At the material level, molecular engineering can be employed to precisely tailor the band structure and surface states of nanomaterials, enabling the design of quantum units with intrinsic coherence protection, such as through topological safeguards. At the system level, external field control techniques, such as dynamical decoupling sequences, can be applied to actively counteract environmental noise in real time. Moreover, drawing inspiration from natural quantum biological processes such as photosynthesis, which employs protein scaffolds and vibrational coupling to prolong electronic coherence times, will provide crucial biomimetic insights for the design of artificial systems.
2. **Designing intelligent quantum biomaterials with integrated biocompatibility and functional controllability**. Quantum biomaterials must achieve an optimal balance among biocompatibility, precise functional controllability, and quantum‑effect activity. This necessitates the implementation of a multidimensional synergistic philosophy in material design. From a chemical perspective, advancing surface functionalization and biomimetic coating is essential to ensure stability, biocompatibility, and avoidance of nonspecific protein adsorption and immune clearance in physiological environments. From a physical perspective, precise engineering of quantum properties such as exciton energy, spin states, and plasmon resonance frequencies enables controlled state switching and functional modulation via external stimuli like light, magnetic fields, electricity, or heat. This integrated design further allows materials to sense microenvironmental biochemical signals, such as pH or enzyme activity, and execute responsive actions, thereby advancing the development of intelligent diagnostic and therapeutic systems.
3. **Establishing a predictable regulatory system linking quantum effects to macroscopic physiological outcomes**. This requires constructing a multi‐scale causal chain that connects nanoscale quantum events to cellular functions and ultimately to tissue‐ and organ‐level physiological responses. This necessitates the development of cross‐scale theoretical and computational models. Quantum chemistry and molecular dynamics simulations can be used to precisely calculate the impact of quantum effects on biomolecular structures, energy transfer, and reaction kinetics. Building on this, systems biology and quantitative cellular models can predict how quantum interventions perturb metabolic networks, signaling pathways, and gene expression. Finally, integrating pharmacokinetic/pharmacodynamic models with data from organoid or in vivo experiments enables the evaluation of overall physiological outputs.
4. **Achieving high‐resolution, real‐time characterization of relevant quantum effects in vivo**. Conventional imaging techniques struggle to directly observe transient and weak quantum events within complex living environments. Overcoming this bottleneck relies on advancing novel quantum sensing and imaging technologies. Quantum optical techniques, such as quantum correlation imaging, offer the potential to dynamically track quantum dot or plasmon resonance energies. The integration of cutting‐edge quantum technologies with multimodal clinical imaging is essential to construct a multi‐layered, real‐time, and dynamic panoramic view that spans from molecular quantum events to overall anatomical structures, which is key to validating and optimizing quantum intervention strategies.

## Conflicts of Interest

The authors declare no conflicts of interest.

## Data Availability

The data that support the findings of this study are available from the corresponding author upon reasonable request.
